# Acute care pathways for patients calling the out-of-hours services

**DOI:** 10.1186/s12913-020-4994-0

**Published:** 2020-02-27

**Authors:** Morten Breinholt Søvsø, Linda Huibers, Bodil Hammer Bech, Helle Collatz Christensen, Morten Bondo Christensen, Erika Frischknecht Christensen

**Affiliations:** 1 Department of Clinical Medicine, Centre for Prehospital and Emergency Research, Aalborg University, Søndre Skovvej 15, 9000 Aalborg, Denmark; 2 Research Unit for General Practice, Aarhus University, Aarhus, Denmark; 3 Department of Public Health, Research Unit for Epidemiology, Aarhus University, Aarhus, Denmark; 4 Emergency Medical Services, Copenhagen, University of Copenhagen, Copenhagen, Denmark

**Keywords:** Out-of-hours medical care, Delivery of health care, Primary care, Emergency medical services, Denmark, Diagnoses, Telephone hotline

## Abstract

**Background:**

In Western countries, patients with acute illness or injury out-of-hours (OOH) can call either emergency medical services (EMS) for emergencies or primary care services (OOH-PC) in less urgent situations. Callers initially choose which service to contact; whether this choice reflect the intended differences in urgency and severity is unknown. Hospital diagnoses and admission rates following an OOH service contact could elucidate this. We aimed to investigate and compare the prevalence of patient contacts, subsequent hospital contacts, and the age-related pattern of hospital diagnoses following an out-of-hours contact to EMS or OOH-PC services in Denmark.

**Methods:**

Population-based observational cohort study including patients from two Danish regions with contact to EMS or OOH-PC in 2016. Hospital contacts were defined as short (< 24 h) or admissions (≥24 h) on the date of OOH service contact. Both regions have EMS, whereas the North Denmark Region has a general practitioner cooperative (GPC) as OOH-PC service and the Capital Region of Copenhagen the Medical Helpline 1813 (MH-1813), together representing all Danish OOH service types. Calling an OOH service is mandatory prior to a hospital contact outside office hours.

**Results:**

OOH-PC handled 91% (1,107,297) of all contacts (1,219,963). Subsequent hospital contacts were most frequent for EMS contacts (46–54%) followed by MH-1813 (41%) and GPC contacts (9%). EMS had more admissions (52–56%) than OOH-PC. For both EMS and OOH-PC, short hospital contacts often concerned *injuries* (32–63%) and *non-specific diagnoses* (20–45%). The proportion of *circulatory disease* was almost twice as large following EMS (13–17%) compared to OOH-PC (7–9%) in admitted patients, whereas *respiratory diseases* (11–14%), *injuries* (15–22%) and *non-specific symptoms* (22–29%) were more equally distributed. Generally, admitted patients were older.

**Conclusions:**

EMS contacts were fewer, but with a higher percentage of hospital contacts, admissions and prevalence of *circulatory diseases* compared to OOH-PC, perhaps indicating that patients more often contact EMS in case of severe disease. However, hospital diagnoses only elucidate severity of diseases to some extent, and other measures of severity could be considered in future studies. Moreover, the socio-demographic pattern of patients calling OOH needs exploration as this may play an important role in choice of entrance.

## Background

In Western countries, patients experiencing acute illness or injury out-of-hours (OOH) can call two types of services: emergency medical services (EMS) [1] in case of emergencies or out-of-hours primary care (OOH-PC) [2] in less urgent situations. The scope of these services is intended to be complementary with EMS handling major injuries and life or limb threatening diseases and OOH-PC handling less acute patients with medical diseases or injuries that cannot wait till the next workday. Most countries have a national emergency number for EMS available to patients, whereas different models exist for provision of other forms of urgent care that can be freely accessible or use telephone triage to manage access [2].

In Denmark, the OOH services consist of a nationwide EMS and two different types of OOH-PC – the general practitioner cooperatives (GPC) in four of five regions and the Medical Helpline 1813 (MH-1813) in the Capital Region of Copenhagen only. They all perform telephone triage and calling is mandatory prior to further health care access. EMS is similarly organized nationwide, whereas OOH-PC have different organizations. The patient or bystander makes the initial choice of whom to contact for help. Due to patient help seeking behaviour and limitations of telephone triage [3–5], patient populations of both services may overlap, i.e. patients in need of emergency care are seen by services intended for less urgent medical situations and vice-versa. Most studies investigating help seeking are based on the involved health care personnel’s assessment of the medical relevance of the choice [6, 7]. The need for hospital contact, especially hospital admission, is a marker of the severity of the condition. Whether patients choose the OOH service most relevant for their condition could be investigated using diagnostic patterns and admission rates from hospitals as surrogate measures for severity and urgency. Thus, making it possible to include a large study cohort. More insight into the proportion of subsequent hospital contacts and disease patterns in terms of hospital diagnoses for patients contacting OOH services could identify if pathways reflect the intended differences in aims. Thus, we aimed to investigate and compare the prevalence of patient contacts, subsequent hospital contacts, and the age-related pattern of hospital diagnoses following an out-of-hours contact to EMS or OOH-PC services in Denmark.

## Methods

### Study design and population

We conducted a population-based observational cohort study from January 1st 2016 to December 31st 2016 of patients from two Danish regions (North Denmark Region and Capital Region of Copenhagen) with contact to EMS or OOH-PC, especially focusing on contacts with subsequent hospital contact. The two regions were chosen to include all types of services existing in Denmark. We only included patients with valid personal identification number (PIN) and residence in the same region as the OOH service investigated.

### Setting

The North Denmark Region is a both rural and urban region with 586,000 inhabitants, whereas the Capital Region of Copenhagen is densely populated (1,789,000 inhabitants) [8]. To access hospital care in Denmark, it is mandatory for patients to call either OOH-PC or EMS. The regions have different OOH-PC services (GPC in the North Denmark Region and MH-1813 in the Capital Region of Copenhagen), but similar EMS organizations. GPs answer all calls at the GPC, performing triage and assessing the adequate response (i.e. telephone advice, consultation, home visit or direct referral to hospital) [9]. At MH-1813, nurses (for the most part) and physicians answer the telephone to decide whether the patient is in need of a telephone advice, consultation, a home visit, or a direct referral to the hospital [10]. The nurses use a computerized decision support tool, when performing telephone triage [11]. MH-1813 carry out home visits and cannot triage patients to an OOH GP consultation, thus face-to-face consultations take place in various hospital emergency departments. Here hospital clinicians (employed by the hospitals) perform the consultations, which are registered as hospital contacts. Emergency medicine is a very new specialty in Denmark, so not many emergency physicians are employed at the hospitals yet [12]. The hospital clinicians that evaluate patients are therefore of various specialties including family medicine. Emergency calls to the national emergency number 1–1-2 are forwarded to the EMS, if of medical nature. Primarily nurses answer the calls. A criteria-based dispatch protocol is used to assess the urgency and severity of the situation and the adequate response (i.e. ambulances, paramedics in rapid response vehicles, doctor, advice using a computerized decision support tool or in some cases the criteria-based dispatch protocol or by conferring with physician). The basic ambulance in Denmark is staffed by two ambulance professionals, of which at least one is at paramedic level or higher. The other ambulance professional may be an ambulance assistant or paramedic. Paramedics with special competencies also man rapid response vehicles as separate entities. Moreover there are Mobile Emergency Care Units (cars) and Helicopter Emergency Services with anesthesiologists [13, 14]. In this study, we considered OOH as 4 P.M to 8 A.M on workdays and all hours on weekends and public holidays (GPC hours) to have comparable data, since EMS and MH-1813 are available 24 h. Danish health care is tax-financed and free of charge, including the EMS and OOH-PC services.

### Data sources and outcome measures

Data on EMS and OOH-PC service contacts was retrieved from the prehospital databases and the National Health Service Registry [15]. We used each citizen’s unique 10-digit PIN [15] for linkage to national registries. Age was obtained through PIN linkage to Statistics Denmark [16]. From the Danish National Patient Registry [17], we identified hospital contacts. We examined if a service had been contacted on the same date as the start date of a hospital contact and if so, which service(s): GPC, MH-1813 or EMS only or both the EMS and one of the OOH-PC services (referred to as *multiple contacts* from hereon). Our outcome measures were: 1) prevalence of OOH service use and 2) subsequent hospital contacts (short hospital contacts < 24 h and hospital admission ≥24 h). Furthermore, we included the final diagnoses received during the hospital contact following an OOH service contact and reported the most frequent subcategory diagnoses stratified by OOH service. For each OOH service and in relation to patient age, we reported the chapter level diagnoses according to the International Statistical Classification of Diseases and Health related Problems 10th Revision (ICD-10) [18]. ICD-10 diagnoses from the chapters 18 (*symptoms and signs*) and 21 (*other factors*) are referred to as *non-specific diagnoses* from hereon. We followed the STROBE guidelines when reporting our results [19].

### Statistical analysis

Data were anonymized for statistical analysis. All reported results are OOH patient contacts with valid PIN and shown together with the total activity (all hours) and total activity with valid PINs in Table 1. Descriptive statistics were used for reporting frequency of contacts to OOH services and subsequent diagnoses in hospital. Most frequent subcategory diagnoses stratified by OOH service and hospital contact type were also reported. We calculated incidence rate ratios (IRR) with 95% confidence intervals (CI), when comparing contact rates. Multiple contacts on the date of hospital contact were reported separately. Diagnostic pattern was reported in relation to patient age. We performed Wilcoxon rank sum test to compare age across OOH service populations. Results are presented with standard deviation (SD), 95%CIs or *p*-values. Statistical analyses were performed with Stata V.15.0/MP (Stata Corporation, College Station, Texas, USA).

**Table 1:** Contact frequency per 1,000 inhabitants (total number)

Health care service	EMS		OOH-PC		Total
	North	Copenhagen	GPC	MH-1813	
All activities, all hours^a^	102 (59,880)	173 (310,907)	560 (328,151)	507 (907,101)	(1,606,309)
Valid PIN, all hours	90 (53,123)	156 (279,393)	560 (328,151)	490 (877,280)	(1,537,947)
Out-of-hours^b^	39 (22,592)	50 (90,074)	560 (328,151)	435 (779,146)	(1,219,963)
Subsequent hospital contacts^c^	21 (12,544)	23 (41,993)	52 (30,307)	178 (319,358)	(404,202)
- Short hospital contacts	10 (5,679)	10 (18,618)	29 (16,867)	144 (258,392)	(299,556)
- Admissions	11 (6,865)	13 (23,375)	23 (13,440)	34 (60,966)	(104,646)

^a^Activities during all hours including OOH shown

^b^Only including EMS contacts related to emergency (1-1-2) calls

^c^Not including patients with multiple contacts

## Results

### Patient pathways

**Fig. 1:**
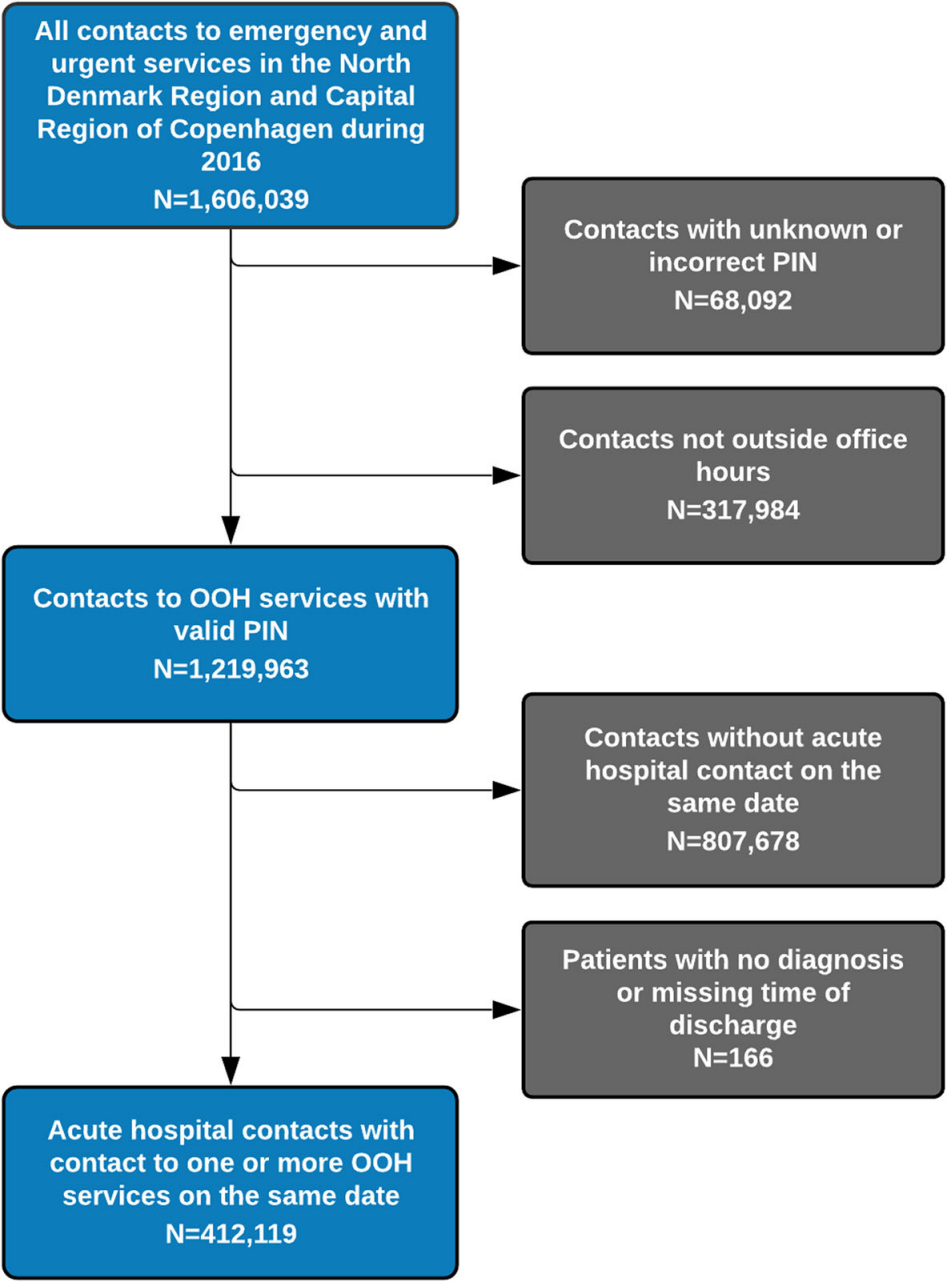
Flowchart showing the inclusion of the study population

In 2016, EMS and OOH-PC services had 1,219,963 OOH patient contacts with valid PIN in the North Denmark Region (North) and Capital Region of Copenhagen (Copenhagen) (Fig. 1) and contacts to OOH-PC comprised 91%. PIN was incorrect or missing in around 10% of EMS contacts and 3% of MH-1813 contacts. GPC had the most contacts per inhabitants of all services (560 per 1000). Activities during OOH and all hours are displayed in Table 1. EMS had the highest percentage of subsequent hospital contacts (54% (North) and 46% (Copenhagen)) followed by contacts MH-1813 (41%) and lastly GPC (9%). Multiple contacts amounted to 7917 cases in total.

### Short hospital contacts

Short hospital contacts were defined as hospital stays with a duration of less than 24 h. Thus, short hospital contact also included face-to-face clinical consultations at the hospital. Following EMS contacts, short hospital contacts constituted 10 per 1000 inhabitants across both regions (IRR = 0.93 (95%CI: 0.90–0.96)). For OOH-PC, the number of short hospital contacts varied and was five times as high for patients contacting the MH-1813 compared to patients contacting the GPC (144 vs. 29 contacts per 1000 inhabitants (IRR = 5.02 (95%CI: 4.94–5.10) (Table 1)). Patients with multiple contacts on the date of a short hospital contact were few in both regions (North (2 per 1000), Copenhagen (1 per 1000) (IRR = 1.44 (95%CI = 1.34–1.56)) (not in table)).

### Hospital admissions

Hospital admissions after EMS contact were comparable in the North and Copenhagen (11 vs. 13 per 1000) (IRR = 0.89 (95%CI: 0.87–0.92)), corresponding to 26–30% of EMS contacts. In comparison, 4–8% of OOH-PC contacts were admitted. As with short contacts, hospital admissions were less frequent for GPC patients compared to MH-1813 patients (23 vs. 34 per 1000) (IRR = 0.67 (95%CI: 0.66–0.69)). Admissions following multiple contacts amounted to 3 per 1000 in the North and 2 per 1000 in Copenhagen (IRR = 1.78 (95%CI: 1.68–1.89)) (not in table).

**Fig. 2:**
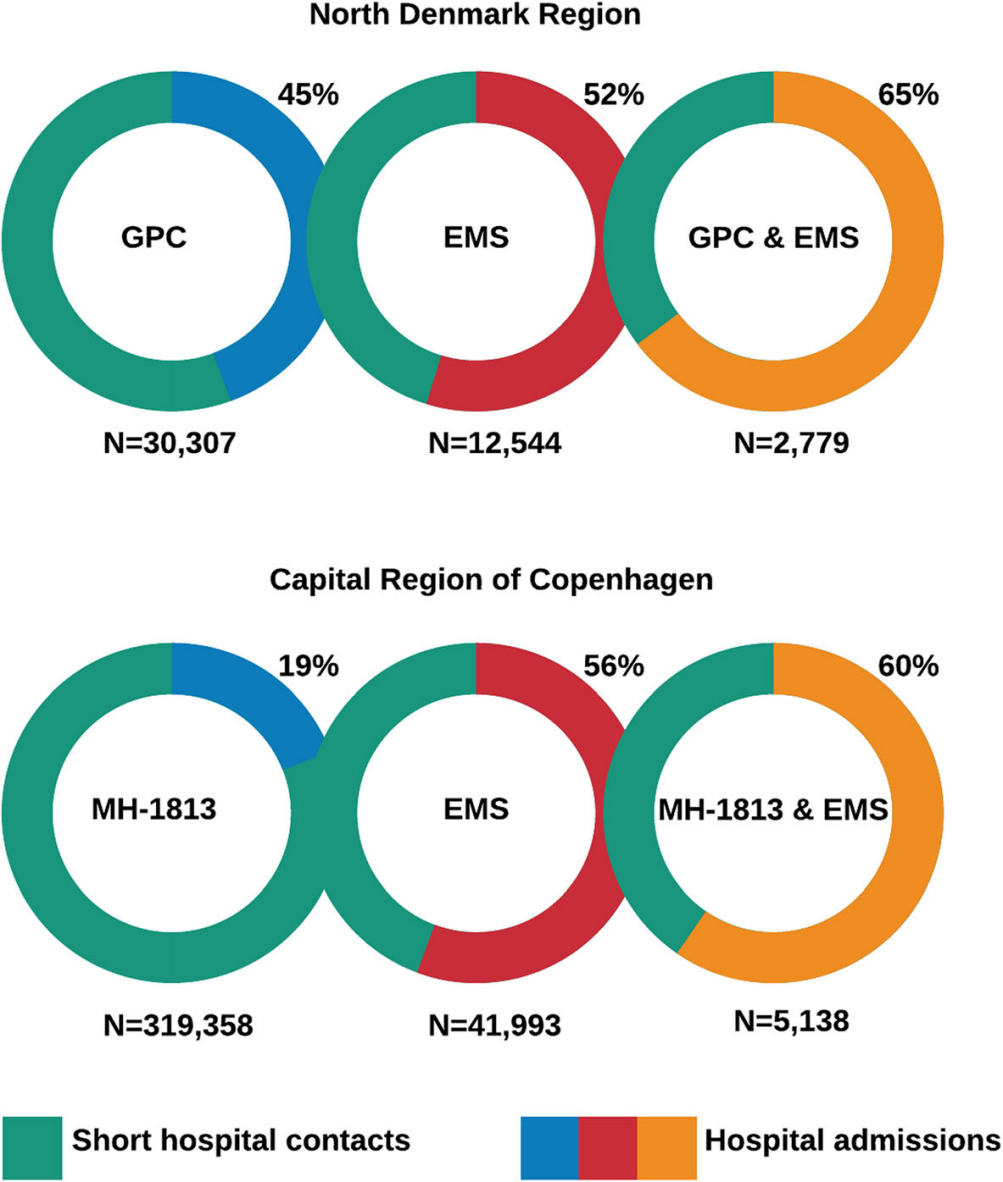
Proportion of subsequent short hospital contact and hospital admission per region and OOH service (*N* = 412,119)

The distribution of short hospital contacts and hospital admissions for each service are shown in Fig. 2.

### Pattern of diagnoses in short hospital contacts

**Fig. 3:**
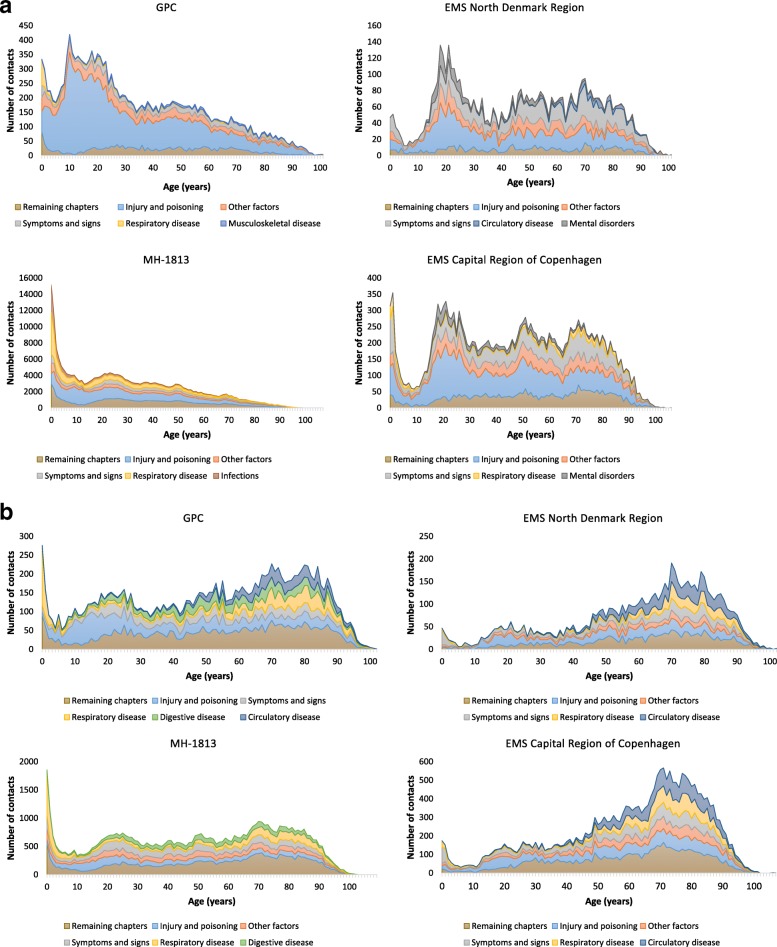
**a** Top 5 of diagnostic pattern (chapters) for short hospital contacts (number of) following GPC, MH-1813 and EMS contacts for age (*N* = 299,556). **b** Top 5 of diagnostic pattern (chapters) for hospital admissions (number of) following GPC, MH-1813 and EMS contact for age (*N* = 104,646)

After both EMS and OOH-PC contacts *injury and poisoning* was the dominating chapter in short hospital contacts (Fig. 3a) and the most frequent subcategory diagnoses within the chapter were minor trauma or injury (additional file 1). EMS proportions of *non-specific diagnoses* (i.e. *symptoms and signs* & *other factors*) (North 44.9% and Copenhagen 36.8%) were almost double those of OOH-PC (MH-1813 19.8% and GPC 22.2%). *Mental disorders* were frequent only after EMS contacts. Age-related patterns were somewhat similar for patients who had an EMS or GPC contact prior to the hospital contact, although the GPC patient population included fewer elderly patients (*p* < 0.00). Short hospital contacts after a MH-1813 contact were dominated by children 4 years and younger (MH-1813 17.5%, GPC 7.6%, EMS North 3.3%, EMS Copenhagen 5.8%) and diagnoses within the chapter *respiratory diseases* (MH-1813 15.7%, GPC 2.3%, EMS North 2.4% and EMS Copenhagen 4.4%). MH-1813 patients were significantly younger than both GPC and EMS patients (*p* < 0.00). Patients with multiple contacts prior to a short hospital contact (3058) were older than OOH-PC and EMS patients (*p* < 0.00) (additional file 2) and mainly received diagnoses regarding *injuries* (27.8 and 18.3%) and to a higher degree *non-specific diagnoses* (56.6 and 35.8%).

### Pattern of diagnoses in hospital admissions

In admissions following both EMS and GPC contacts, the age and diagnostic patterns were more homogenous, changing towards an elderly population (often older than 60–65 years) (Fig. 3b, additional file 2) with a wider range of diagnostic chapters, but EMS patients were still older (*p* < 0.00). The *non-specific diagnoses* and diagnoses from the chapters *respiratory disease* and *injury and poisoning* were frequent both among EMS patients and OOH-PC patients. *Circulatory disease* was almost twice as frequent following EMS contacts (13–17%) compared to OOH-PC contacts (7 and 9%). At subcategory level, the most frequent diagnosis regarding circulatory disease in EMS contacts was *cerebral infarction, unspecified* (additional file 1). The opposite was the case for *digestive diseases*. As with short hospital contacts, admissions following MH-1813 contacts more often concerned children 4 years and below (7.5%) than the other OOH services (range 2.0–4.9%) and overall MH-1813 patients were younger (*p* < 0.00).

Admitted patients with multiple contacts (4859) were older than both OOH-PC and EMS patients (*p* = 0.00) (additional file 2). In the North, they most frequently received diagnoses from the *circulatory disease* chapter (21.1%), whereas diagnoses concerning *respiratory disease* were most frequent in the Copenhagen (17.3%). The overall distribution of diagnostic chapters within each OOH service can be seen in Table 2.

**Table 2:** Most frequent ICD-10 chapters for short hospital contacts and hospital admissions per OOH service (percent) (sorted by overall ICD-10 chapters contributing more than 1%)

Short hospital contacts					
	All	EMS		OOH-PC	
ICD-10 chapter	% *N* = 299,556	North *N* = 5679	Copenhagen *N* = 18,618	GPC *N* = 16,867	MH-1813 *N* = 258,392
Injury and poisoning	33.9	33.7	37.0	62.6	31.8
Respiratory disease	14.0	2.4	4.4	2.3	15.7
Symptoms and signs	11.4	29.6	22.1	8.8	10.4
Other factors	10.1	15.3	14.7	13.4	9.4
Infections	7.3	0.5	1.4	1.2	8.2
Genitourinary disease	4.9	1.2	1.9	1.2	5.4
Musculoskeletal disease	3.7	2.5	2.6	2.1	3.9
Skin disease	3.2	0.2	0.4	0.8	3.6
Ear disease	3.0	0.3	0.4	0.3	3.4
Eye disease	2.8	0.2	0.2	1.9	3.1
Digestive disease	2.5	1.2	2.3	1.5	2.6
Circulatory disease	1.1	3.4	3.6	1.7	0.9
Remaining chapters	2.1	9.9	9.0	2.2	1.6
Total	100	100	100	100	100
Hospital admissions					
	All	EMS		OOH-PC	
ICD-10 chapter	% *N* = 104,646	North *N* = 6865	Copenhagen *N* = 23,375	GPC *N* = 13,440	MH-1813 *N* = 60,966
Injury and poisoning	17.2	21.6	19.8	22.2	14.7
Symptoms and signs	15.9	17.1	15.3	13.7	16.5
Respiratory disease	13.1	10.7	12.1	12.8	13.9
Other factors	11.4	9.8	11.5	7.4	12.4
Circulatory disease	9.0	16.8	13.2	8.7	6.6
Digestive disease	8.6	5.6	5.4	11.7	9.4
Infections	5.3	2.6	3.0	5.1	6.5
Genitourinary disease	4.6	2.4	2.5	5.0	5.6
Neurological disease	2.8	3.7	4.6	1.9	2.1
Musculoskeletal disease	2.7	1.4	1.6	2.3	3.3
Endocrine disease	2.6	1.9	2.7	2.9	2.6
Mental disorders	2.1	3.5	4.0	1.6	1.3
Skin disease	1.4	0.2	0.3	1.2	1.9
Pregnancy & childbirth	1.1	0.6	1.9	0.9	0.8
Remaining chapters	2.2	2.1	2.1	2.6	2.1
Total	100	100	100	100	100

## Discussion

### Key results

The EMS handled 9% of all acute contacts, while OOH-PC handled 91%. Subsequent hospital contacts were most frequent for EMS contacts followed by MH-1813. Following both EMS and OOH-PC contacts short hospital contacts often involved younger patients and often concerning *injury* diagnoses. *Non-specific diagnoses* were very frequent, constituting more than one fifth of all short hospital contacts and more than one third after EMS contacts. Short hospital contacts following a MH-1813 contact often concerned children 4 years and below and *respiratory diseases*. Among admitted patients, *circulatory disease* was almost twice as frequent following EMS contacts compared to OOH-PC. Admissions after EMS and OOH-PC contact were often older patients and showed substantial overlap in diagnoses, often concerning *respiratory disease, injuries* and *non-specific symptoms*. Although few, patients with multiple contacts were most frequently admitted and often with *circulatory* and *respiratory disease.*

### Strengths and weaknesses of the study

The population-based design included every admission as well as every registered OOH service contact with PIN available from the selected regions, therefore minimizing selection bias and resulting in a large cohort. We linked OOH service contacts to hospital contacts by PIN and date. Doing so may have resulted in a smaller population size, since patients who had an OOH service contact before midnight may have had a hospital contact after midnight. Moreover, linking by date could have introduced a selection bias, as diagnostic patterns may differ during day and night [20–22]. Most OOH service contacts occur during the afternoon and early evening, and we suspect the loss of patients to be minor [20, 23]. The unique PIN allowed for linking patient contact with other registries and databases. Although the validity of the Danish National Patient Registry is relatively high [17], no other clinical data was obtained to verify the diagnoses or the severity of these. We had complete follow-up on the included patients, but in some contacts the PIN was not registered - predominately in the EMS setting (Table 1). This may result in underestimation of the number of subsequent hospital contacts since it was not possible to link to any hospital contact without the correct PIN. The missing PINs are a known problem at EMS, mostly for the least urgent calls, which constitute around 15–20% [14, 23] and studies have shown missing PIN in 18% of all calls [21] and 47% in the least urgent calls [22]. Least urgent calls probably represent patients who are least likely to have a hospital contact as shown by Lehm et al., who found that 60% of least urgent calls by patients with correct PINs had no further contact to the health care system within 1 day of an EMS call [22]. The missing PINs have probably affected our results regarding the pattern of diseases, most likely for short hospital contacts due to the low urgency. Furthermore, the inhabitants of the two regions included have important differences in socioeconomic and demographic characteristics such as lower income, lower education and higher age in the North Denmark Region compared to the Capital Region of Copenhagen [24]. A study also showed higher mortality in the North Denmark Region following a cardiovascular event [25]. These differences have most likely affected our results – especially since younger patients have a different diagnostic pattern compared to older patients. Our study might have reported too few hospital contacts, because the implementation of a new electronic medical record system in the hospitals in the Capital Region of Copenhagen led to fewer reports to the Danish National Patient Registry. Lastly, we primarily used diagnoses at chapter level when reporting disease patterns. Chapters include diagnoses of both high and low urgency and severity and cannot stand alone as a measure of severity. To address this limitation, we included subcategory diagnoses and admissions rates in our assessment of severity.

### Comparison with literature

As found in this study, Denmark has a relatively high number of OOH-PC contacts per inhabitants (approximately 500/1000) [11, 26] compared with other countries with similar health care organizations (ranging from around 150–410/1000) [27–29], whereas the level of EMS contacts (100/1000) is more alike [1, 30, 31]. In the present study, the number of subsequent hospital contacts depending on type of OOH service differed, which was to be expected when comparing EMS and OOH-PC. However, the two OOH-PC services included showed substantial differences, most likely owing to the fact that patients who contact MH-1813 triaged to clinic consultations, get their consultation at the hospital, therefore registered as a hospital contact. Telephone triage at MH-1813 is performed by nurses using a computerized decision support tool, which may lead to more clinic consultations compared to triage by GPs [32]. However, we interpret the large difference in short hospital contacts as a consequence of the fact that face-to-face consultation are performed at hospitals, since no OOH consultations by GPs are possible and not as meaning that the triaging nurses were referring a vastly higher proportion of patients to hospital.

This study reported hospital contacts and diagnoses following OOH service contacts, which may differ from daytime hospital contacts/all acute hospital contacts. Emergency calls to EMS display a diurnal pattern with the highest number of call occurring during daytime [23] and differences in proportion of certain diseases and admission rates between daytime and OOH in primary care have also been reported [33–35]. Nevertheless, Vest-Hansen et al. [36] reported *circulatory disease* (19.3%), *other factors* (16.9%)*, infections* (15.5%)*, symptoms and signs* (11.8%) and *injury and poisoning* (6.3%) as the top five ICD-10 chapters used in acute admissions to a medical ward (not including surgical specialties, which explains the lower proportion of *injuries and poisoning*) and a study on emergency department contacts found *injuries and poisoning* (38.3%)*, symptoms and signs* (16.1%)*, other factors* (14.5%)*, circulatory diseases* (5.7%) and *respiratory diseases* (5.4%) as the most frequent chapters [37]. These studies did not investigate whether the patients arrived at hospital after calling EMS or OOH-PC. However, it has previously been shown that a large proportion of patients brought to hospital after ambulance transport by EMS receive a broad range of diagnoses including non-urgent and/or non-specific diagnoses [21, 38, 39] and that a substantial proportion of patients with serious conditions such as myocardial infarction or stroke initially contact primary care (both during daytime and OOH) [40–44]. This could indicate some overlap in patient populations. Two of these studies also included 30-day mortality rates for ICD-10 chapters and found that *circulatory disease* were among the chapters with the highest mortality rates ranging from 7.5 to 14.7% [21, 37]. Blinkenberg et al. [35] investigated the referring professional of hospital admissions (GP, OOH doctor, outpatient clinic/private specialist and direct admission) and the diagnostic pattern in a nationwide Norwegian study. In good agreement with ours and other studies, the study reported *injuries and poisoning, circulatory disease, symptoms and signs, respiratory disease* and *digestive disease* (in that order) as the most frequent diagnostic chapters used for admissions (the chapter *other factors* was excluded from the study).

### Interpretation

Our results showed that during out-of-hours, 91% of all patient contacts are handled by OOH-PC. Thus, changes within the organization of OOH-PC services may have great impact on patient contacts to hospital. We also found that EMS contacts more often resulted in admissions, than OOH-PC contacts, which could indicate more severe disease in line with the higher prevalence of *circulatory disease* among EMS contacts. Our results indicate some overlap in diagnostic pattern and age, mainly for admitted patients. We found only few patients with multiple contacts, but as they were admitted frequently and often with severe conditions, they represent an important and vulnerable patient group who might be ‘falling through the cracks’. Future research could include patients with multiple contacts and their sociodemographic characteristics.

## Conclusion

To summarize, EMS contacts were fewer, but with a higher percentage of hospital contacts, admissions and prevalence of *circulatory diseases* compared to OOH-PC. This may indicate that patients more often contact EMS in case of severe disease. However, hospital diagnoses only elucidate severity of diseases to some extent, and other measures of severity could be considered in future studies. Moreover, the socio-demographic pattern of patients calling OOH needs exploration as this may play an important role in choice of entrance.

## Supplementary information

**Additional file 1 **Top five most frequent subcategory diagnoses for short hospital contacts and admissions stratified by OOH service. Top five most frequent subcategory diagnoses for short hospital contacts and admissions stratified by OOH service, (%), (*N* = 404,202)

**Additional file 2 **Patient age for short hospital contacts and admissions. Patient age (years) for short hospital contacts and admissions (mean (SD) *N* = 412,119)

## Data Availability

The data that support the findings of this study are available from the North Denmark Region and the Capital Region of Copenhagen, but restrictions apply to the availability of these data, which were used under license from the Danish Patient Safety Authority for the current study, and so are not publicly available.

## Abbreviations

EMS: Emergency Medical Services
GP: General practitioner
GPC: General practitioner cooperatives
ICD-10: International Statistical Classification of Diseases, 10th Edition
MH-1813: Medical Helpline 1813
OOH: Out-of-hours
OOH-PC: Out-of-hours primary care
PIN: Personal identification number
SD: Standard deviation
STROBE: STrengthening the Reporting of Observational Studies in Epidemiology

## References

[1] Langhelle A, Lossius HM, Silfvast T, Björnsson HM, Lippert FK, Ersson A (2004). International EMS systems: the Nordic countries. Resuscitation.

[2] Huibers L, Giesen P, Wensing M, Grol R (2009). Out-of-hours care in western countries: assessment of different organizational models. BMC Health Serv Res.

[3] Keizer E, Smits M, Peters Y, Huibers L, Giesen P, Wensing M (2015). Contacts with out-of-hours primary care for nonurgent problems: patients’ beliefs or deficiencies in healthcare?. BMC Fam Pract.

[4] Huibers L, Keizer E, Carlsen AH, Moth G, Smits M, Senn O (2018). Help-seeking behaviour outside office hours in Denmark, the Netherlands and Switzerland: a questionnaire study exploring responses to hypothetical cases. BMJ Open.

[5] Huibers L, Smits M, Renaud V, Giesen P, Wensing M (2011). Safety of telephone triage in out-of-hours care: a systematic review. Scand J Prim Health Care.

[6] Nørøxe KB, Huibers L, Moth G, Vedsted P. Medical appropriateness of adult calls to Danish out-of-hours primary care: a questionnaire-based survey. BMC Fam Pract. 2017:1–9. Available from: https://www.ncbi.nlm.nih.gov/pmc/articles/PMC5351208/. Accessed 19 Feb 2020. 10.1186/s12875-017-0617-1 PMC5351208 28292257

[7] Vilke G, Anthony E (2011). Factors associated with ambulance use among emergency department patients. Acad Emerg Med.

[8] Statistics Denmark. Danmarks Statistik [Statistics Denmark]. Available from: http://www.statistikbanken.dk/statbank5a/default.asp?w=1440. Cited 2020 Jan 14

[9] Olesen F, Jolleys JV (1994). Out of hours service: the Danish solution examined. BMJ.

[10] Region Hovedstaden (2013). Afrapportering af akut og præhospital indsats i Region Hovedstaden [Report on acute and prehospital efforts in the Capital Region].

[11] Graversen DS, Pedersen AF, Carlsen AH, Bro F, Huibers L, Christensen MB (2019). Quality of out-of-hours telephone triage by general practitioners and nurses: development and testing of the AQTT–an assessment tool measuring communication, patient safety and efficiency. Scand J Prim Health Care.

[12] Sundhedsstyrelsen, Dansk Selskab for Akutmedicin (2018). Målbeskrivelse for speciallægeuddannelsen i Akutmedicin [Program Description for specialist training in Emergency Medicine].

[13] Danske Regioner (2014). Dansk Indeks for Akuthjælp [Danish index for emergency care].

[14] Andersen MS, Johnsen SP, Sørensen JN, Jepsen SB, Hansen JB, Christensen EF (2013). Implementing a nationwide criteria-based emergency medical dispatch system: A register-based follow-up study. Scand J Trauma Resusc Emerg Med.

[15] Andersen JS, Olivarius NDF, Krasnik A (2011). The Danish National Health Service Register. Scand J Public Health.

[16] Statistics Denmark. About Us - Statistics Denmark. Available from: http://www.dst.dk/en/OmDS#. Cited 2020 Jan 14.

[17] Schmidt M, Schmidt SAJ, Sandegaard JL, Ehrenstein V, Pedersen L, Sørensen HT. The Danish National Patient Registry: a review of content, data quality, and research potential. Clin Epidemiol. 2015;7:449. Available from: https://www.dovepress.com/the-danish-national-patient-registry-a-review-of-content-data-quality--peer-reviewed-article-CLEP 10.2147/CLEP.S91125 PMC4655913 26604824

[18] World Health Organization (2016). International Statistical Classification of Diseases and Related Health Problems 10th Revision.

[19] von Elm E, Altman DG, Egger M, Pocock SJ, Gøtzsche PC, Vandenbroucke JP (2014). The Strengthening the Reporting of Observational Studies in Epidemiology (STROBE) Statement: Guidelines for reporting observational studies. Int J Surg.

[20] Moth G, Flarup L, Christensen MB, Olesen F, Vedsted P (2011). Kontakt- og sygdomsmønsteret i lægevagten LV-KOS 2011 [Contact and disease pattern in the GPC LV-KOS 2011].

[21] Christensen EF, Larsen TM, Jensen FB, Bendtsen MD, Hansen PA, Johnsen SP (2016). Diagnosis and mortality in prehospital emergency patients transported to hospital: a population-based and registry-based cohort study. BMJ Open.

[22] Lehm KK, Andersen MS, Riddervold IS (2017). Non-urgent emergency callers: characteristics and prognosis. Prehosp Emerg Care.

[23] Møller TP, Ersbøll AK, Tolstrup JS, Østergaard D, Viereck S, Overton J (2015). Why and when citizens call for emergency help: an observational study of 211,193 medical emergency calls. Scand J Trauma Resusc Emerg Med.

[24] Henriksen DP, Rasmussen L, Hansen MR, Hallas J, Pottegård A (2015). Comparison of the five Danish regions regarding demographic characteristics, healthcare utilization, and medication use - A descriptive cross-sectional study. PLoS One.

[25] Kjærulff TM, Bihrmann K, Zhao J, Exeter D, Gislason G, Larsen ML (2019). Acute myocardial infarction: does survival depend on geographical location and social background?. Eur J Prev Cardiol.

[26] Ebert JF, Huibers L, Christensen B, Lippert FK, Christensen MB (2019). Giving callers the option to bypass the telephone waiting line in out-of-hours services: a comparative intervention study. Scand J Prim Health Care.

[27] Huibers L, Moth G, Andersen M, van Grunsven P, Giesen P, Christensen MB (2014). Consumption in out-of-hours health care: Danes double Dutch?. Scand J Prim Health Care.

[28] The demand for out-of-hours care from GPs: A review [Internet]. Vol. 17, Family Practice. C. Salisbury, Division of Primary Health Care, University of Bristol, Canynge Hall, Whiteladies Road, Bristol BS8 2PR, United Kingdom: Oxford University Press (Great Clarendon Street, Oxford OX2 6DP, United Kingdom); 2000. p. 340–7. Available from: http://ovidsp.ovid.com/ovidweb.cgi?T=JS&PAGE=reference&D=emed8&NEWS=N&AN=30623822. Accessed 19 Feb 2020. 10.1093/fampra/17.4.340 10934185

[29] Moll Van Charante EP, Van Steenwijk-Opdam PCE, Bindels PJE (2007). Out-of-hours demand for GP care and emergency services: patients’ choices and referrals by general practitioners and ambulance services. BMC Fam Pract.

[30] Lowthian JA, Jolley DJ, Curtis AJ, Currell A, Cameron PA, Stoelwinder JU (2011). The challenges of population ageing: accelerating demand for emergency ambulance services by older patients, 1995-2015. Med J Aust.

[31] Ellensen EN, Hunskaar S, Wisborg T, Zakariassen E (2014). Variations in contact patterns and dispatch guideline adherence between Norwegian emergency medical communication centres--a cross-sectional study. Scand J Trauma Resusc Emerg Med.

[32] Moth G, Huibers L, Vedsted P (2013). From doctor to nurse triage in the Danish out-of-hours primary care service: simulated effects on costs. Int J Family Med.

[33] Johansen IH, Morken T, Hunskaar S (2010). Contacts related to mental illness and substance abuse in primary health care: a cross-sectional study comparing patient’s use of daytime versus out-of-hours primary care in Norway. Scand J Prim Health Care.

[34] Flarup L, Moth G, Christensen MB, Vestergaard M, Olesen F, Vedsted P (2014). Chronic-disease patients and their use of out-of-hours primary health care: A cross-sectional study. BMC Fam Pract.

[35] Blinkenberg J, Pahlavanyali S, Hetlevik Ø, Sandvik H, Hunskaar S (2019). General practitioners’ and out-of-hours doctors’ role as gatekeeper in emergency admissions to somatic hospitals in Norway: registry-based observational study. BMC Health Serv Res.

[36] Vest-Hansen B, Riis AH, Sørensen HT, Christiansen CF (2014). Acute admissions to medical departments in Denmark: diagnoses and patient characteristics. Eur J Intern Med.

[37] Søvsø MB, Hermansen SB, Færk E, Lindskou TA, Ludwig M, Møller JM (2018). Diagnosis and mortality of emergency department patients in the North Denmark region. BMC Health Serv Res.

[38] Snooks H, Williams S, Crouch R, Foster T, Hartley-Sharpe C, Dale J (2002). NHS emergency response to 999 calls: alternatives for cases that are neither life threatening nor serious. BMJ.

[39] Christensen EF, Bendtsen MD, Larsen TM, Jensen FB, Lindskou TA, Holdgaard HO (2017). Trends in diagnostic patterns and mortality in emergency ambulance service patients in 2007−2014: a population-based cohort study from the North Denmark region. BMJ Open.

[40] Doggen CJM, Zwerink M, Droste HM, Brouwers PJAM, van Houwelingen GK, van Eenennaam FL (2016). Prehospital paths and hospital arrival time of patients with acute coronary syndrome or stroke, a prospective observational study. BMC Emerg Med.

[41] Thylén I, Ericsson M, Hellström Ängerud K, Isaksson RM, Sederholm LS (2015). First medical contact in patients with STEMI and its impact on time to diagnosis; an explorative cross-sectional study. BMJ Open.

[42] Faiz KW, Sundseth A, Thommessen B, Rønning OM (2017). Prehospital path in acute stroke. Tidsskr Den Nor Laegeforening Tidsskr Prakt Med Ny Raekke.

[43] Faiz KW, Sundseth A, Thommessen B, Rønning OM (2013). Prehospital delay in acute stroke and TIA. Emerg Med J.

[44] Ellensen E. N., Naess H., Wisborg T., Hunskaar S., Zakariassen E. (2017). Stroke identification by criteria based dispatch - a register based study. Acta Anaesthesiologica Scandinavica.

