# Supplementary material for: Acute care pathways for patients calling the out-of-hours services
Source: BMC Health Serv Res. 2020 Feb 27;20:146. doi: 10.1186/s12913-020-4994-0 (PMC7045402; doi:10.1186/s12913-020-4994-0)
Supplement: Supplementary file 1 — Additional file 1 Top five most frequent subcategory diagnoses for short hospital contacts and admissions stratified by OOH service. Top five most frequent subcategory diagnoses for short hospital contacts and admissions stratified by OOH service, (%), (N = 404,202) [file 12913_2020_4994_MOESM1_ESM.docx]

|  |  | |  |
| --- | --- | --- | --- |
| North Denmark Region | | **Capital Region of Copenhagen** | |
| GPC | **EMS** | **MH-1813** | **EMS** |
|  | **Short hospital contacts** | |  |
| *Sprain and strain of ankle* 6.8% | *Observation for suspected disease or condition, unspecified* **5.1%** | *Viral infection, unspecified* **4.3%** | *Observation for suspected disease or condition, unspecified* 2**.8%** |
| *Observation for suspected disease or condition, unspecified* 3.6% | *Chest pain, unspecified* **3.9%** | *Acute upper respiratory infection, unspecified* **3.7%** | *Acute abdomen* **2.5%** |
| *Persons encountering health services in other specified circumstances* 3.5% | *Concussion* **3.8%** | *Acute cystitis* **3.0%** | *Open wound of scalp* **2.5%** |
| *Fracture of lower end of radius* 2.7% | *Acute intoxication* **3.4%** | *Pneumonia, unspecified* **2.5%** | *Chest pain, unspecified* **2.2%** |
| *Contusion of finger(s) without damage to nail* 2.2% | *Syncope and collapse* **2.2%** | *Acute tonsillitis, unspecified* **2.4%** | *Superficial injury of scalp* **2.0%** |
|  | **Hospital admissions** | |  |
| *Pneumonia, unspecified* 3.8% | *Pneumonia, unspecified* **3.0%** | *Acute abdomen* **4.7%** | *Pneumonia, unspecified* **2.8%** |
| *Acute abdomen* 3.4% | *Cerebral infarction, unspecified* **3.0%** | *Pneumonia, unspecified* **3.6%** | *Acute respiratory failure* **2.1%** |
| *Chronic obstructive pulmonary disease with acute exacerbation, unspecified* 1.9% | *Chronic obstructive pulmonary disease with acute exacerbation, unspecified* **2.9%** | *Observation for suspected disease or condition, unspecified* **2.0%** | *Chronic obstructive pulmonary disease with acute exacerbation, unspecified* **2.1%** |
| *Observation for suspected disease or condition, unspecified* 1.5% | *Syncope and collapse* **2.1%** | *Observation for other suspected diseases and conditions* **1.7%** | *Observation for suspected myocardial infarction* **2.1%** |
| *Cerebral infarction, unspecified* 1.4% | *Observation for suspected disease or condition, unspecified* **1.8%** | *Urinary tract infection, site not specified* **1.4%** | *Cerebral infarction, unspecified* **1.9%** |

**Top five most frequent subcategory diagnoses for short hospital contacts and admissions stratified by OOH service, (%) (N=404,202)**
