# Supplementary material for: Acute care pathways for patients calling the out-of-hours services
Source: BMC Health Serv Res. 2020 Feb 27;20:146. doi: 10.1186/s12913-020-4994-0 (PMC7045402; doi:10.1186/s12913-020-4994-0)
Supplement: Supplementary file 2 — Additional file 2 Patient age for short hospital contacts and admissions. Patient age (years) for short hospital contacts and admissions (mean (SD) N = 412,119) [file 12913_2020_4994_MOESM2_ESM.docx]

**Patient age (years) for short hospital contacts and admissions (mean (SD)) N=412,119**

|  | Short hospital contact | Admission |
| --- | --- | --- |
| EMS |  |  |
| North Denmark Region | 47.0 (24.8) | 60.1 (22.6) |
| Capital Region of Copenhagen | 46.8 (25.4) | 60.5 (22.7) |
| All | **46.8 (25.3)*** | **60.4 (22.7)*^✝^** |
| OOH-PC |  |  |
| GPC | 34.2 (24.1) | 51.5 (26.9) |
| MH-1813 | 30.1 (23.5) | 49.1 (27.6) |
| All | **30.3 (23.5)*** | **49.5 (27.5)*^✝^** |
| Multiple contacts |  |  |
| EMS & GPC | 51.4 (24.3) | 62.6 (21.0) |
| EMS & MH-1813 | 47.4 (26.5) | 64.3 (23.2) |
| All | **48.7 (25.7)*** | **63.7 (22.5)*^✝^** |

*comparison of age between overall groups (EMS, OOH-PC and multiple contacts), p<0.00

**^✝^**comparison of age in short hospital contacts and admissions, p<0.00
